# Bioprocess optimization for lactic and succinic acid production from a pulp and paper industry side stream

**DOI:** 10.3389/fbioe.2023.1176043

**Published:** 2023-05-18

**Authors:** Agata Olszewska-Widdrat, Charilaos Xiros, Anders Wallenius, Roland Schneider, Laís Portugal Rios da Costa Pereira, Joachim Venus

**Affiliations:** ^1^ Microbiome Biotechnology Department, Leibniz Institute for Agricultural Engineering and Bioeconomy (ATB), Potsdam, Germany; ^2^ RISE Processum AB, Örnsköldsvik, Sweden

**Keywords:** fermentation, lactic acid, succinic acid, fibre sludge, hydrolysate, bioeconomy

## Abstract

The effective and cheap production of platform chemicals is a crucial step towards the transition to a bio-based economy. In this work, biotechnological methods using sustainable, cheap, and readily available raw materials bring bio-economy and industrial microbiology together: Microbial production of two platform chemicals is demonstrated [lactic (LA) and succinic acid (SA)] from a non-expensive side stream of pulp and paper industry (fibre sludge) proposing a sustainable way to valorize it towards economically important monomers for bioplastics formation. This work showed a promising new route for their microbial production which can pave the way for new market expectations within the circular economy principles. Fibre sludge was enzymatically hydrolysed for 72 h to generate a glucose rich hydrolysate (100 g·L^−1^ glucose content) to serve as fermentation medium for *Bacillus coagulans* A 541, A162 strains and *Actinobacillus succinogenis* B1, as well as *Basfia succiniciproducens* B2. All microorganisms were investigated in batch fermentations, showing the ability to produce either lactic or succinic acid, respectively. The highest yield and productivities for lactic production were 0.99 g·g^−1^ and 3.75 g·L^−1^·h^−1^ whereas the succinic acid production stabilized at 0.77 g·g^−1^ and 1.16 g·L^−1^·h^−1^.

## 1 Introduction

The chemical production of lactic or succinic acid is facing new challenges, associated with sustainability issues and the usage of fossils for their production. An alternative way to produce them is the biotechnological utilization of residual forms of lignocellulosic biomass as second generation sugar rich feedstocks, including industrial streams such as sulphite fibre sludge, respecting the “food first” principle. The use of affordable, industrial, side streams as feedstock is of great interest to establish circular economy, and to decrease the cost of the final products. Sulphite fibre sludge (SFS) is a residual side stream obtained from pulp mills and biorefineries. It is usually relatively easy to hydrolyse it enzymatically, without prior thermochemical pre-treatment, which is advantageous, as pre-treatment may result in formation of substances that inhibit microbial growth, such as furfural or phenolic compounds (Xie et al., 2018; Ludwig et al., 2013). The annual production of SFS is ∼1000 dry tonnes/paper mill plant, making it a valuable feedstock for value added bio-based products and specialty chemicals. Lactic and succinic acid are two monomers that belong to the group of 12 main platform chemicals (Rodrigues et al., 2017; Alexandri and Venus, 2017; Lu et al., 2021; Yankov, 2022; Rawoof et al., 2021). The market share of biotechnological processes for the production of platform chemicals is expected to increase in the coming years from 5% to 20% (Yankov, 2022). The global lactic acid production is expected to reach 5.8 billion by 2030, according to a new report by Grand View Research, Inc., (Grandviewresearch, 2022), which only shows how the market interest is increasing. The market growth can be assigned with demand for lactic acid in food, beverages, pharma industry, and as a feedstock in the production of poly-lactic acid (PLA), which is going to drive the market globally. Lactic acid contains three carbons, one hydroxyl and the carboxyl group, which makes this molecule extremely attractive in many fields, such as materials science, food packaging, among others (Murali et al., 2017; Ismail et al., 2018; P. Pawar et al., 2014). Lactic acid is often added as pH stabilizer, or to increase taste features of food products. Similarly, succinic acid plays an important role in biomaterials, bio-surfactants, precursor for other chemicals, etc. The succinic acid market is estimated to reach USD 182.8 million by 2023, increasing at a CAGR of 6.8% from 2018 (Malanca et al., 2022). Companies, such as, Biosuccinium, BioAmber, Myriant, or Succinity have been responsible for the main production of succinic acid via microbial fermentation. Unfortunately, due to economic competitiveness, most of the succinic acid is still produced using petrochemical routes (Kuenz et al., 2020). Succinic acid (SA) is a C4 platform molecule, with two carboxyl groups. It serves as an intermediate building block for other chemicals, such as, esters, 1,4-butanediol, itaconic, maleic, aspartic acid, among others (Yang et al., 2020). Succinic acid is also a precursor for polyethylene succinate that has been widely used in the plastic industry. Concerning biotechnological SA production, *Actinobacillus succinogenesis* was considered to be the most promising bacterial strain (Kuenz et al., 2020). Additionally, it was shown that succinic acid can be also produced from lignocellulosic biomass, but more efficient microbial cultivation must be developed to produce it more effectively (Lu et al., 2021). Although, both lactic acid and succinic acid are well known and are widely used in many disciplines, their sustainable production is still developing. Therefore, the decrease in production costs could be achieved by a cheap feedstock and its long-term supply. Additionally, their production from lignocellulose biomass tends to increase, due to the fact, that this type of feedstock does not compete with food and feed sector. Lignocellulose is the most abundant renewable source on Earth, and is mainly composed of cellulose, hemicellulose and lignin (Lu et al., 2021). Lignocellulosic biomass contains a mixture of C6 and C5 sugars. Usually, only C6 sugars are utilized by microbes in fermentation process, but this study shows that C5 sugars, such as xylose, can be successfully converted to organic acids. *Bacillus coagulans* is a spore-forming, facultative anaerobe, thermophilic, able to grow at higher temperatures, above 50°C. It has the ability to convert hexose and pentose sugars to _L_-(+)-lactic acid with high titers (Glaser and Venus, 2018). Therefore, its potential in utilizing lignocellulosic materials is rising. *A. succinogenes* (B1) is able to produce succinic acid anaerobically from a broad range of carbon sources, such as, arabinose, cellobiose, fructose, galactose, glucose, lactose, mannitol, among others (Borges and Pereira, 2011). It is a Gram-negative bacterium, utilizing CO_2_ as an electron acceptor, which induces the metabolism of the substrate to succinate at high CO_2_ levels (Van der Werf et al., 1997). *Basfia succiniciproducens* (B2) was isolated from bovine rumen and described by the German chemical company BASF in Ludwigshafen, Germany (Scholten and Dägele, 2008). This facultative anaerobic microorganism also has a broad substrate utilization spectrum, including lignocellulosic biomass (Ventorino et al., 2017; Bartos et al., 2021). To summarize microorganisms used in this study, this approach presents a possibility to combine production of low-cost feedstock with high-value-added products, such as organic acids within the concept of bio-refinery. Lactic acid production by two strains of *B. coagulans* (A541) and *B. coagulans* (A162) was investigated, whereas succinic acid production was performed by B1 and B2 strains. Both monomers were studied using batch fermentation method. Conversion of SFS to lactic acid was studied with thermotolerant A541 and A 162 strains, whereas succinic acid production was performed at 30°C.

## 2 Materials and methods

### 2.1 Substrate

Sulphite Fibre sludge (SFS), a side stream of the pulp and paper industry was used as feedstock. SFS was enzymatically hydrolysed to generate a hydrolysate rich in glucose which was used as the substrate for lactic and succinic acid production. The enzymatic hydrolysis was performed in 50 L hydrolysis reactors (Belach, Sweden) equipped with stirring, temperature and pH control, specifically designed for high solids handling. Hydrolysis was performed at 50°C, pH5, and 100 rpm using the commercial cellulolytic cocktail (Cellic CTec2, Novozymes, Bagsvaerd, Denmark). The SFS loading was 15% (on dry basis). The reaction mixture had a total mass of 50 kg. The duration of the hydrolysis was 72 h. After hydrolysis, the pH of the hydrolysate was adjusted to 3 with sulphuric acid. The hydrolysate was then sterile-filtered and stored at 4°C. The composition of all batches delivered is shown in Table 2, whereas the composition after the enzymatic hydrolysis is shown in Table 2.

The study of the enzyme dosage in SFS hydrolysis was performed in 100 ml Erlenmeyer flasks in a shaking incubator (180 rpm, 50°C) with 150 g·L^−1^ of dry SFS as the substrate. The experiments were carried out in duplicate.

### 2.2 Screening of microorganisms

After the screening of more than 200 lactic acid producing strains, which mostly come from the collection of the Leibniz Institute for Agricultural Engineering and Bioeconomy (ATB, Potsdam, Germany), two candidates were chosen for further batch experiments. A541 and A162 strains were used for lactic acid production. A screening was necessary in order to evaluate, if SFS is sufficient for the growth of 200 *B. coagulans* isolates. The screening criteria were based on the preference of the microorganism in glucose, cellobiose, xylose, and arabinose utilization. Before the screening, all microbial strains were reactivated from cryo-stock in 5 mL in Man, Rogosa and Sharpe (MRS) medium. pH of fibre sludge hydrolysate was firstly adjusted to 6.4 with 4% (w/v) NaOH and sterilized at 121°C, for 15 min. SFS with 15 g·L^−1^ of yeast extract was prepared in the same way. For control experiments the solution with 100 g·L^−1^ of glucose with 15 g·L^−1^ of yeast extract, with pH adjusted to 6.5 with 4% (w/v) NaOH was sterilized at 121°C, for 15 min. Screening was carried out using Bioscreen C (Growth Curves United States) equipment, where optical density (OD) is measured in a microplate. A volume of 250 µL of either hydrolysate or glucose and 20 µL of pre-culture was used per each well. Duplicate measurements were done at 5 min intervals for each strain. The overall duration of the bioscreen was 48 h.

### 2.3 Microorganisms and inoculum preparation

*B. coagulans* A541 strain was isolated from olive residues, whereas A162 was isolated from potato waste water. Both strains were characterized using matrix assisted laser desorption/ionization time-of-flight mass spectrometry (MADI-ToF MS) method. Both strains are L-(+)-LA producers. Strains are available at the ATB collection and are stored at—80°C, as a cryo-stock. The inoculum was prepared in MRS bouillon (Merck, with dolomite EVERZIT (0.5–2.5 mm; Evers, Germany) as buffering system, at an orbital shaker at 100 rpm, temp. set at 40°C, 15 h.

*A. succinogenes* B1 and *B. succiniciproducens* B2 were used for succinic acid production. B1 strain (DSM—22251) was obtained from DSMZ (Braunschweig, Germany) was used in experiments carried out at the ATB. B2 strain (DSM—22022) was purchased from the University of Bern and used in experiments carried out at the ATB. The inocula were prepared in the same way for both strains, were CASO bouillon was used with two cryo beads, incubated at 37°C in shaking flasks, at 150 rpm, for 20 h.

### 2.4 Lab-scale fermentation set-up

Lactic acid fermentations were performed in small scale bioreactors. Fermentations were carried out, in duplicate, with 250 mL working volume, at 52°C, and 200 rpm, using Eloferm multi-fermentation system (Biotronix GmbH, Germany). The pH was stabilized at pH 6.0 with 20% (w/v) NaOH solution. The inoculum size was 2% (v/v) and samples were taken every 2 h for sugars and LA quantification. The medium was supplemented with 10 g·L^−1^ of yeast extract. Sulphite fibre sludge was used as a carbon source at the amount of 100 g·L^−1^. Yields were calculated by means of g LA per g of total sugars.

Succinic acid fermentations were also performed in small scale bioreactors, in duplicate, with 250 mL working volume. Temp. was set at 37°C, and steering speed at 200 rpm, for 20 h. The pH was stabilized at 6.7 with 25% Na_2_CO_3_ solution (w/v). The inoculum size was 10% (v/v) and samples were taken every 2 h for sugars and succinic acid quantification. The medium was supplemented with 15 g·L^−1^ of yeast extract. Similarly, sulphite fibre sludge was used as a carbon source at the amount of 100 g·L^−1^. Yields were calculated by means of g SA per g of total sugars.

### 2.5 Analytics

High performance liquid chromatography—HPLC - (DIONEX, Sunnyvale, CA, United States) was used to detect the sugar content and organic acids concentration. It was coupled with a refractive index detector (RI-71, SHODEX, Yokohama, Japan) and equipped with a Eurokat H column (300 mm × 8 mm × 10 μm; Knauer, Germany), eluted with 5 mM H_2_SO_4_ at 0.8 mL·min^−1^. Cation analysis in the hydrolysate were performed by using an IonPac CS 16 column (250 mm × 4 μm, DIONEX, Sunnyvale, CA, United States), operating at flow rate of 1.0 mL·min^−1^, at 40°C, with 30 mM CH_3_SO_3_H as mobile phase. Anion analysis was carried out utilizing IonPac AS9-HC column (250 mm × 4 μm, DIONEX; Sunnyvale, CA; United States), eluted with Na_2_CO_3_ at a flow rate of 1.2 mL·min^−1^, at room temperature. Furfural, hydroxymethylfurfural and phenols were evaluated in a Dionex ICS 3000 system (Thermo Fisher Scientific) equipped with an Eurospher II 100-5 C18 with precolumn (Knauer, Germany) connected to a UV-visible detector (280 nm) using ultrapure water and 50% acetonitrile at 1 mL·min^−1^. Ethanol was analysed using gas chromatography (GC) system (7890A, Agilent Technologies) equipped with DB-WAX Ultra Inert column (30 m × 0.25 mm length; d.f.: 0.25 µm) and a flame ionization detector (FID) at 300°C.

## 3 Results

### 3.1 Sulphite fibre sludge (SFS) hydrolysis and characterization

The enzyme loadings for the hydrolysis of SFS were studied regarding the released glucose. Enzyme loadings in the range of 0.02–0.1 g Enz·g^−1^ DM were studied corresponding to 2.6–13.2 FPU·g^−1^ DM. As shown in Table 1 released glucose increases as enzyme loading increases, however, glucose concentration is not proportionally increased as shown by the rate of glucose released/enzyme loading. However, the enzyme loading of 0.1 g Enz·g^−1^ FSF was selected for the pilot scale experiments due to the need for elevated initial glucose concentration during the fermentation step.

**TABLE 1:** Glucose release from SFS applying different enzyme loadings. The values obtained are averages from duplicate reactions (50°C, 180 rpm) and standard errors were below 5% in all cases.

FSF loading (D.M g·L^−1^)	Enzyme loading (g _Enz_·g^−1^_FSF_)	Enzyme loading (FPU·g^−1^_FSF_)	Glucose (g·L^−1^, 72 h)	Glucose (g·g^−1^_Enz_)
150	0.02	2.6	44.0	15
150	0.05	6.6	67.7	9
150	0.075	9.9	88.6	8
150	0.1	13.2	94.1	6

The SFS hydrolysate used in the fermentation experiments (produced in 50 L hydrolysis reactor) consisted of glucose, cellobiose, and xylose, with glucose being the predominant sugar with the concentration of around 100 g·L^−1^, for all batches, as shown in Table 1. The SFS contained also small amounts of lactic acid, and acetic acid, ethanol. It also contained nitrogen, phosphorus that could serve as nutrient source in lactic and succinic acid fermentations. It also contained small amounts of inhibitors, such as furfural, HMF and phenols, but these amounts did not affect the growth of microbial strains.

### 3.2 Screening

**FIGURE 1:**
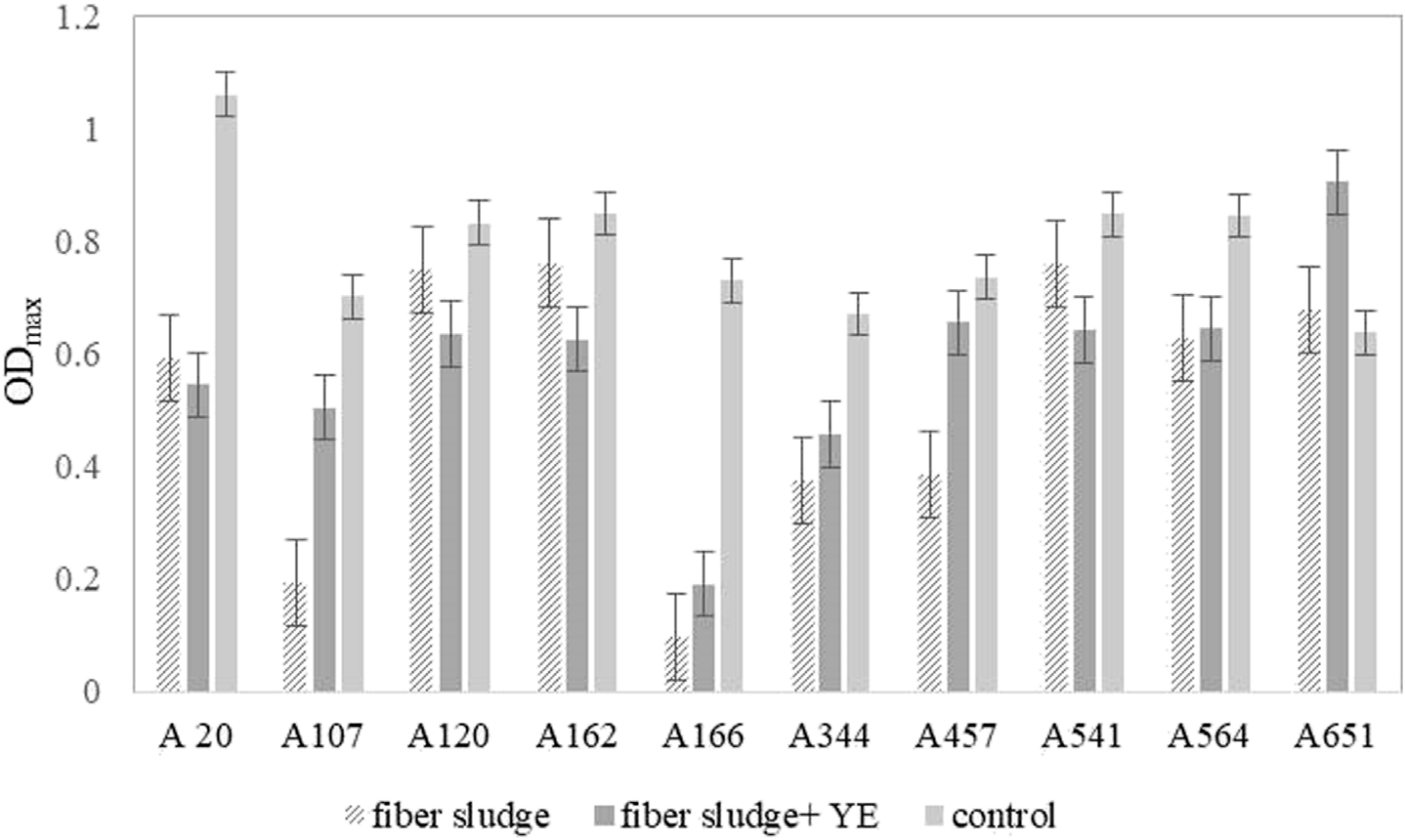
Maximal optical density (OD_max_) achieved in a microplate reader by 10 different *B. coagulans* strains. Results show the average of duplicates. These strains are 10 representative isolates. Other 190 tested strains are shown in Supplementary Information.

Results obtained during the screening, shown in Figure 1, indicate that SFS could serve as a good substrate for further microbial fermentations. This small scale, preliminary step was necessary to exclude strains that would be sensitive to inhibitors present in SFS, or different ratio of sugars could affect in bigger or smaller scale their behaviour. Therefore, this step was crucial to determine, if the growth of the strain could be supported by this new hydrolysate. Based on the origin of the strain and previous experience, two strains A162 and A541, both homofermentative L-LA producers, were chosen for batch fermentations (López-Gómez et al., 2019; Glaser and Venus, 2018).

### 3.3 Effect of different carbon sources on LA production

**FIGURE 2:**
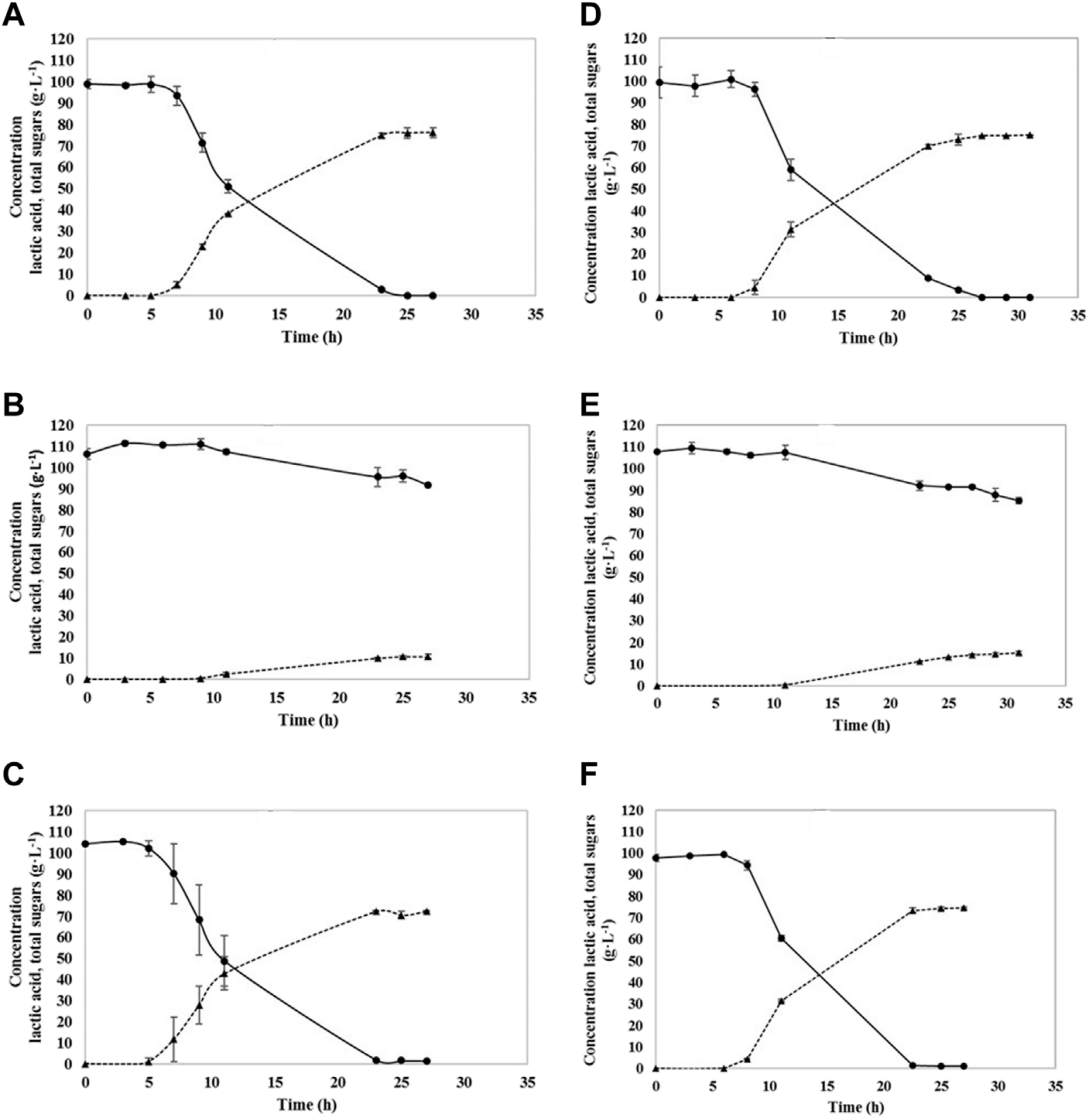
Sugar consumption and lactic acid production utilizing A541 **(A–C)** and A162 **(D–F)**; glucose + YE **(A,D)**, hydrolysate only **(B,E)**, and hydrolysate + YE **(C,F)**. Lactic acid (▲), Total sugars (●).

The fermentation with the YE supplementation exhibited faster growth and higher LA concentrations, what can be seen in Figure 2. Glucose was fully consumed by A541 and A162 after 23 and 27 h, respectively. When fiber sludge hydrolysate was utilized, almost all sugars were consumed. In the case of A541, only 1.38 g·L^−1^ of cellobiose remained unconsumed, whereas fermentation with A162 resulted in 1.08 g·L^−1^ of unconsumed cellobiose. Utilizing the hydrolysate had a positive effect on lactic acid production. Both strains, A162 and A541 produced the highest amount of LA, 79.1 g·L^−1^ and 77.2 g·L^−1^, showing the highest productivities of: 3.50 and 3.58 g·L^−1^·h^−1^. All yields calculated were above 0.9 g·g^−1^ when YE were used, with the highest 0.98 g·g^−1^ for A162 strain utilizing SFS. By-products were not formed during fermentations. Fermentation was finished after 23–25 h.

### 3.4 Effect of different carbon sources on SA production

**FIGURE 3:**
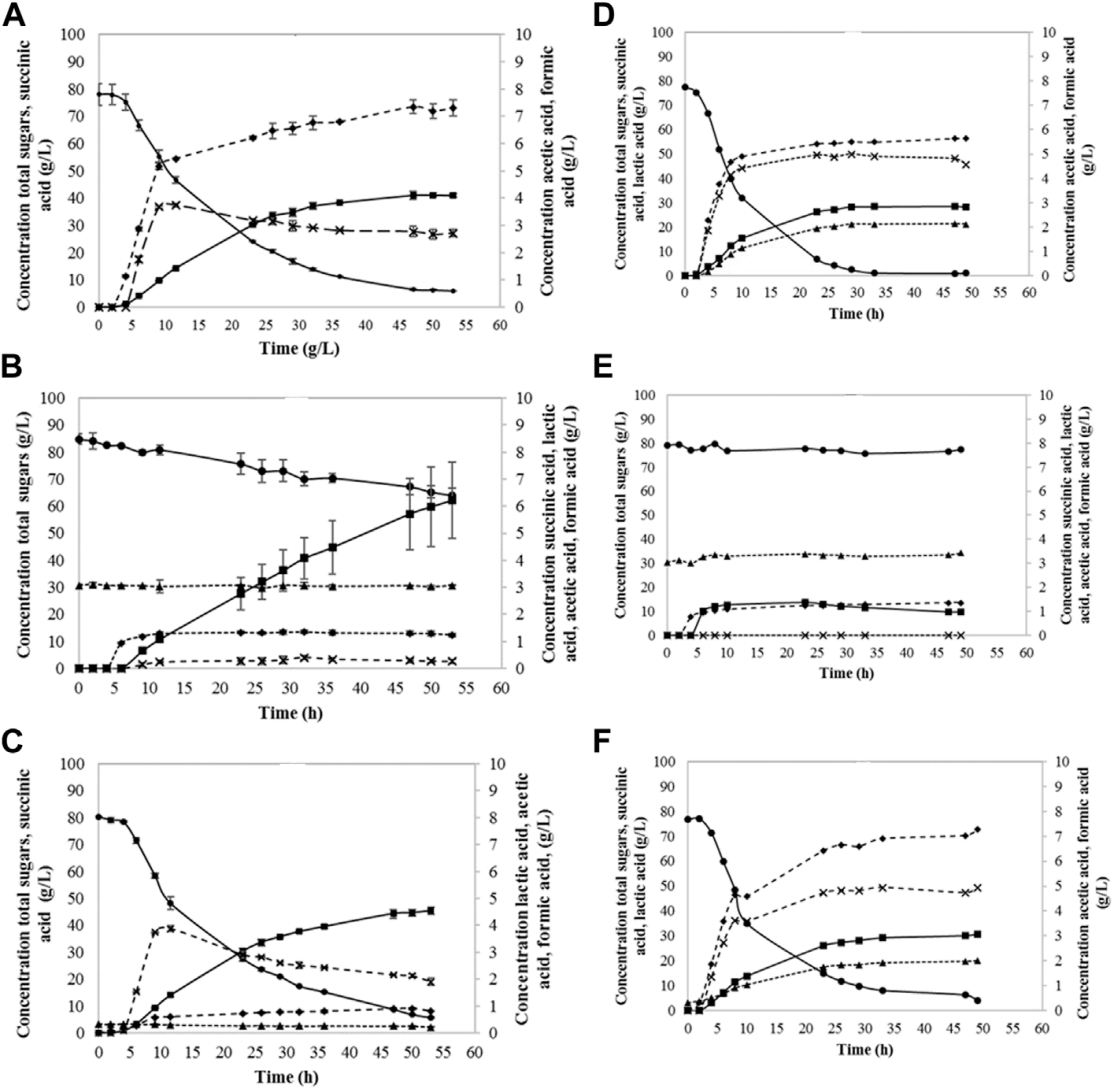
Sugar consumption and succinic acid production utilizing B1 strain **(A–C)** and B2 strain **(D–F)**: glucose + YE **(A,D)**, pure hydrolysate **(B,E)**, and hydrolysate + YE **(C,F)**. Lactic acid (▲), total sugars (●), succinic acid (■), acetic acid (♦), formic acid (x).

Succinic acid was successfully produced from fibre sludge by both B1 and B2 strain (shown in Figure 3). The control experiments with glucose resulted in succinic acid formation at the concentration between 30—40 g·L^−1^, indicating that *A. succinogenes* was performing better, showing higher yields and productivities of 0.7 g·g^−1^ and 0.79 g·L^−1^·h^−1^, respectively. Batch fermentations without YE showed a very slow growth for both strains, and accordingly very low succinic acid production, which indicated that yeast extract had a strong effect on the performance of both strains. When synthetic glucose was applied, both strains were able to metabolize glucose, but still, some sugars remained unconsumed. The highest amount of succinic acid was produced while utilizing SFS and *A. succinogenes* B1, where 45.4 g·L^−1^ of SA was produced, with yield of 0.77 g·g^−1^ and productivity of 0.96 g·L^−1^·h^−1^. B2 strain showed slightly lower yield of 0.52 g·g^−1^ and productivity of 0.63 g·L^−1^·h^−1^, resulting in 30.6 g·L^−1^ of SA produced. During SA fermentation, by-product formation occurred, were acetic and formic acid were also detected.

## 4 Discussion

In this study, sulphite fibre sludge (SFS) hydrolysate was used as the feedstock and carbon source for lactic and succinic acid production. Notably, SA and LA yields were higher when SFS was used, comparing to control fermentations with glucose, what is summarized in Table 3. That shows that all strains prefer a mixture of C5 and C6 sugars for a sustainable growth, which is consistent with previous studies on batch fermentation of single sugar (Ferone et al., 2017). SFS contains a mixture of C5 and C6 sugars, with glucose as a predominant sugar. Cellobiose and xylose were detected in this stream as well. In addition, a mixture of phosphorus and nitrogen, or other ions, such as magnesium, or potassium was detected which likely enabled strains for a better nutrient provision and in consequence for better performance.

Lactic acid fermentation, to be economically feasible, requires low-cost feedstock. Therefore, SFS was one of the choices concerning not only this aspect, but also serving as a valuable carbon source. Its low inhibitor content enabled the growth of *B. coagulans* strains without any difference, comparing to standard, glucose medium. Moreover, it enhanced the growth of LA producing bacteria, thanks to additional nutrients present in the hydrolysate that can be seen in Table 2. Lactic acid production from lignocellulosic feedstock, has been already reported and summarized by (Yankov, 2022), emphasizing that cellulosic biomass is an abundant and sustainable source for organic acids production. Therefore, the next step is required in the development of industrial lactic acid production via biotechnological routes, meaning the final shift from oil to renewable resources. As shown in this study, *B. coagulans* strains were able to grow and consume sugars achieving almost 100% conversion to lactic acid, with yields of 0.98 g·g^−1^ and 0.92 g·g^−1^ for both strains used. Food waste was also a potential biomass for lactic acid production (López-Gómez et al., 2019), but recently, the interest in the usage of lignocellulosic waste has been further increasing. Several studies utilizing corn stover (Yi et al., 2016), oil palm empty fruit bunch (Ye et al., 2014), rice straw (Kuo Cheng et al., 2015), among others have been reported.

**TABLE 2:** Composition of SFS after enzymatic hydrolysis. All values are in g·L^−1^. Analysis methods are described in Section 3.1.

Component	Batch 1	Batch 2	Batch 3
Glucose (g·L^−1^)	100	99.7	98.7
Cellobiose (g·L^−1^)	3.34	3.15	4.04
Xylose (g·L^−1^)	1.69	1.70	1.35
Lactic acid (g·L^−1^)	6.60	6.56	4.07
Acetic acid (g·L^−1^)	0.76	0.70	0.45
Ethanol (g·L^−1^)	0.45	0.29	0.12
Total nitrogen (mg·L^−1^)	190	188	201
Total phosphorus (mg·L^−1^)	15.8	15.8	16.7
Cl^−^ (mg·L^−1^)	36.0	35.1	15.5
SO_4_^2+^ (mg·L^−1^)	152	150	213
Na^+^ (mg·L^−1^)	1910.0	1868.0	1334.0
K^+^ (mg·L^−1^)	19.5	19.4	32.0
Mg^2+^ (mg·L^−1^)	16.6	16.9	17.7
Ca^2+^ (mg·L^−1^)	14.6	14.1	14.7
Furfural (mg·L^−1^)	2.04	1.58	<0.0124
HMF (mg·L^−1^)	<0.004	<0.004	<0.004
Phenol (mg·L^−1^)	<0.079	<0.079	<0.079
Catechol (mg·L^−1^)	15.5	15.1	<0.027

In SA batch fermentation, a lag phase was observed that lasted less than 5 h and the exponential growth phase was coupled with fast consumption of sugars and production of succinic acid. However, two other acids acetic acid (AA) and formic acid (FA) were detected. After about 40 h, the base consumption stopped, which indicated that the cell growth also stopped. At this point fermentations were kept running due to unconsumed sugars, but after 50 h were terminated. The final concentration of SA reached 45.4 g·L^−1^ utilizing the B1 strain. The summary of all titers, productivities, and yields, shown in Table 3. Although *B. succiniciproducens* showed the ability to metabolize a wide spectrum of carbon sources, *A. succinogenes* was able to convert sugars effectively. This could be related to the natural habitat of B1 strain, where the variety of carbon sources is very broad. Comparing other wild type microorganisms able to produce SA, reported in this study (Jiang et al., 2017), B1 strain showed similar behaviour to strains reported. Only one strain, CGMCC 1593, was able to produce 60.2 g·L^−1^ of SA from glucose (Yu-Peng et al., 2008). *B. succiniciproducens* was used in succinic acid production to develop a cost-competitive bioprocesses with respect to the formulation of low cost and efficient fermentation medium (Stylianou et al., 2021). But among other microorganisms, *A. succinogenes* is recognized as one of the most promising candidates for industrial production of succinic acid (Carvalho et al., 2016). In another study, *A. succinogenes* was able to utilize SPS, but it was also shown that this strain can utilize straw (Pu et al., 2009), or even sugarcane bagasse hemicellulose hydrolysate (Borges and Pereira, 2011), among others. By-product formation could be related to the CO_2_ source. In other studies utilizing MgCO_3_ (Guettler et al., 1996) showed that MgCO_3_ could have an influence on the metabolic pathway. Therefore, other studies, utilizing other CO_2_ sources are necessary to reduce the amount of by-products formation. On the other hand, Carvalho et al. used fed-batch system, utilizing carob pods and *A. succinogenes* in order to reduce by-product formation (Carvalho et al., 2016).

**TABLE 3:** Yield, global productivity, final acid concentration, and the amount of residual sugars for LA and SA producing strains.

Conditions	Y_LA_ (g/g)	Pg_LA_ (g/L*h)	LA (g/L)	Residual sugars	Y_SA_ (g/g)	Pg_SA_ (g/L*h)	SA (g/L)	Residual sugars
Glucose + YE								
A162	0.93	2.80	75.0	0.0	—	—	—-	—
A541	0.95	3.18	76.2	0.0	—	—	—-	—
B1	—	—	—-	—	0.70	0.99	41.0	5.95
B2	—	—	—-	—	0.48	0.93	28.3	1.01
Fiber sludge hydrolysate								
A162	0.22	0.49	19.7	85.2	—	—	—-	—
A541	0.17	0.39	15.0	91.79	—	—	—-	—
B1	—	—	—-	—	0.09	0.12	6.22	64.08
B2	—	—	—-	—	0.02	0.02	0.98	77.4
Hydrolysate + YE								
A162	0.98	3.50	79.1	1.08	—	—	—-	—
A541	0.92	3.58	77.2	1.38	—	—	—-	—
B1	—	—	—-	—	0.77	0.96	45.4	5.71
B2	—	—	—-	—	0.52	0.63	30.6	4.04

To conclude, fiber sludge serves as a cheap, but reach in simple sugars, that enable a successful fermentation to LA and SA. Since SFS can be produced at quantities varying from 1000 tonnes to 2000 tonnes per plant per year (info from the fibre sludge provider), depending on the size of the plant and the production process, it is regarded as an excellent way to valorize the side stream for the pulp and paper industry, if the process is integrated in the pulp mill. It also enables to perform further studies on technical and pilot scale. This work performed here paves the way for the valorization of more side streams from pulp and paper industry.

## Data Availability

The datasets presented in this study can be found in online repositories. The names of the repository/repositories and accession number(s) can be found below: https://doi.org/10.5281/zenodo.7674351.
